# Effect of a musical intervention on tolerance and efficacy of non-invasive ventilation in the ICU: study protocol for a randomized controlled trial (MUSique pour l’Insuffisance Respiratoire Aigue - Mus-IRA)

**DOI:** 10.1186/s13063-016-1574-z

**Published:** 2016-09-13

**Authors:** Jonathan Messika, David Hajage, Nataly Panneckoucke, Serge Villard, Yolaine Martin, Emilie Renard, Annie Blivet, Jean Reignier, Natacha Maquigneau, Annabelle Stoclin, Christelle Puechberty, Stéphane Guétin, Aline Dechanet, Amandine Fauquembergue, Stéphane Gaudry, Didier Dreyfuss, Jean-Damien Ricard

**Affiliations:** 1 AP-HP, Hôpital Louis Mourier, Réanimation Médico-chirurgicale, 178 rue des Renouillers, F-92700 Colombes, France; 2 Université Paris Diderot, Sorbonne Paris Cité, IAME, UMRS 1137, F-75018 Paris, France; 3 INSERM, IAME, U1137, F-75018 Paris, France; 4 Université Paris Diderot, Sorbonne Paris Cité, ECEVE, UMRS 1123, F-75010 Paris, France; 5 INSERM, ECEVE, U1123, F-75010 Paris, France; 6 INSERM, CIC-EC 1425, UMR 1123, F-75010 Paris, France; 7 APHP, Hôpital Louis Mourier, Département d’Epidémiologie et Recherche Clinique, 178 Rue des Renouillers, Colombes, F-92700 France; 8 Université Paris Diderot, UMR 1123, Sorbonne Paris Cité, Paris, France; 9 Centre Hospitalier Départemental de Vendée, Réanimation Médico-Chirurgicale, La Roche-sur-Yon, F-85925 Cedex 9 France; 10 Institut Gustave Roussy, Réanimation Médico-chirurgicale, Villejuif, F-94800 France; 11 CHRU de Montpellier, Service de Neurologie, Inserm U1061, Montpellier, F-34000 France; 12 Present address: Réanimation Médico-chirurgicale, Hôpital Louis Mourier, 178 rue des Renouillers, F-92700 Colombes, France

**Keywords:** Non-invasive ventilation, Critical care, Music intervention, Respiratory comfort

## Abstract

**Background:**

Non-invasive ventilation (NIV) tolerance is a key factor of NIV success. Hence, numerous sedative pharmacological or non-pharmacological strategies have been assessed to improve NIV tolerance. Music therapy in various health care settings has shown beneficial effects. In invasively ventilated critical care patients, encouraging results of music therapy on physiological parameters, anxiety, and agitation have been reported. We hypothesize that a musical intervention improves NIV tolerance in comparison to conventional care. We therefore question the potential benefit of a receptive music session administered to patients by trained caregivers (“musical intervention”) to enhance acceptance and tolerance of NIV.

**Methods/design:**

We conduct a prospective, three-center, open-label, three-arm randomized trial involving patients in the intensive care unit (ICU) who require NIV, as assessed by the treating physician. Participants are allocated to a “musical intervention” arm (“musical intervention” applied during all NIV sessions), to a “sensory deprivation” arm (sight and hearing isolation during all NIV sessions), or to the control group. The primary endpoint is the change in respiratory comfort (measured with a digital visual scale) before the initiation and after 30 minutes of the first NIV session. The evaluation of the primary endpoint is performed blindly from the treatment group. Secondary endpoints include changes in respiratory and cardiovascular parameters during NIV sessions, the percentage of patients requiring endotracheal intubation, day-90 anxiety/depression and health-related quality of life, post-trauma stress induced by NIV, and the overall assessment of NIV.

The follow-up for each participant is 90 days. We expect to randomize a total of 99 participants.

**Discussion:**

As music intervention is a simple and easy-to-implement non-pharmacological technique, efficacious in reducing anxiety in critically ill patients, it appeared logical to assess its efficacy in NIV, one of the most stressful techniques used in the ICU. Patient centeredness was crucial in choosing the outcomes assessed.

**Trial registration:**

ClinicalTrials.gov: NCT02265458. Registered on 25 August 2014.

**Electronic supplementary material:**

The online version of this article (doi:10.1186/s13063-016-1574-z) contains supplementary material, which is available to authorized users.

## Background

Acute respiratory failure (ARF) is the most frequent organ failure in patients in the intensive care unit (ICU) [1]. Among the various respiratory supports, non-invasive ventilation (NIV) allows the administration of a positive pressure of air and oxygen, at a set inspiratory flow and FiO_2_, which unloads respiratory muscles and enables alveolar recruitment. NIV has shown its efficacy in acute or chronic respiratory failure [2] and cardiac pulmonary edema [3] in terms of intubation rates [4, 5], mortality [5], and nosocomial infections [6]. These positive findings have not been so clearly found in de novo ARF, where NIV benefits may be outweighed by the risks of delaying intubation [7, 8], which has been associated with increased mortality [8]. Although NIV offers many undisputable advantages over invasive ventilation, its intolerance is one of its major drawbacks. Also, premature cessation of NIV because of intolerance may lead to intubation in some cases [7–9]. Hence, investigators have searched for means of improving tolerance of the technique by increasing patient comfort. Light sedation has been shown to decrease NIV-induced discomfort [10–13] and appears to be safe and effective, but this alternative remains a pharmacologic intervention requiring the administration of potentially dangerous anesthetic drugs, such as propofol, remifentanil, or dexmedetomidine. Among non-pharmacologic interventions, sophrology, which aims at increasing well-being through differential relaxation, has been assessed with interesting results [14]. However, only a limited number of patients have been evaluated, and the major drawback is the need for patient adherence, a specific caregiver’s training, and the presence of a single person with the patient for an entire 30-minute session, which makes it very dependent on the availability of the team.

Music therapy in health care settings has recently been investigated. In the perioperative setting, it has shown beneficial effects on anxiety, pain, analgesia use, and patient satisfaction [15]. In the ICU, several studies [16–19] and a recent meta-analysis [20] described its effects in terms of anxiety and physiological changes (heart rate, respiratory rate, and blood pressure) in patients on invasive mechanical ventilation. These studies showed that music therapy is feasible in the ICU, with beneficial effects on physiological parameters, anxiety, and agitation.

### Study rationale

As anxiety is one of the major components of NIV discomfort that leads to NIV cessation, we therefore questioned the potential benefit of music therapy as a non-pharmocological adjunct to NIV to enhance acceptance and tolerance of the technique.

As "music therapy" implies the intervention of a qualified music therapist, we choose to use the term “musical intervention,” which refers to a receptive music session administered to a patient by trained caregivers. To distinguish the potential effect of an isolation from the ICU’s noise and light nuisance, we decided to build a three-arm trial, as performed in invasively ventilated ICU subjects by Chlan et al. [17].

### Study objectives

The primary objective of the present trial is to assess the hypothesis that a musical intervention administered to patients with ARF in the ICU improves NIV tolerance and thereby their comfort and ventilation parameters after 30 minutes of NIV, in comparison to conventional care.

The secondary objectives are to assess musical intervention effect on respiratory comfort and respiratory and cardiovascular parameters during NIV sessions; on NIV failure; on anxiety and agitation during NIV; anxiety/depression and health-related quality of life; post-trauma stress induced by NIV; and on overall assessment of NIV in terms of discomfort, trauma, and satisfaction.

## Methods/design

### Design

Mus-IRA (MUSique pour l’Insuffisance Respiratoire Aigue) is a prospective, multicenter, open-label, three-arm randomized trial. This study is conducted in ICU patients with ARF requiring NIV. Participants are allocated either to an interventional arm or to the control group. A musical intervention is applied during NIV sessions in the first intervention arm. Participants included in the second intervention arm undergo sight and hearing isolation (sensory deprivation). Participants included in the control arm receive conventional treatment associated with NIV.

### Definitions

“Non-invasive ventilation” refers to a bilevel positive pressure ventilation applied via a non-invasive interface (a face mask); “music intervention” refers to a receptive music session administered to a patient by trained caregivers; “respiratory comfort” refers to a “0” rate on a 10-cm dyspnea visual analog scale; “NIV intolerance” refers to the need for the premature cessation of an NIV session; “NIV failure” is defined by the need of endotracheal intubation.

### Ethical aspects

The ethics committee of the French Society for Intensive Care Medicine (Commission d’Ethique de la Société de Réanimation de Langue Française approval CE SRLF 14–21) and the competent French authorities (Comité de Protection des Personnes d’Île de France IV, Hôpital Saint-Louis; registration number 2014-A00643-44; date of approval 26 March 2015) approved the study protocol and patient information documents. According to French law on interventional studies, the patient’s (or his next of kin’s) written informed consent is mandatory for inclusion and randomization.

### Participating ICUs

The three French participating units (Louis Mourier, Gustave Roussy, and CHD de Vendée) have an expertise in NIV. Nurses and nursing assistants have received music intervention training by a skilled music therapist. All participating ICU staff members have received training in the study procedures and protocols to provide the musical intervention and standardized NIV sessions.

### Study population

Eligible patients are adults (≥18 years of age), admitted to the ICU for ARF or exhibiting an ARF during their ICU stay, for whom the physician in charge considers that NIV is indicated.

In each participating ICU, NIV-treated patients are screened for eligibility by the staff members (investigators) 24 hours a day, 7 days a week.

### Inclusion and exclusion criteria

The inclusion criteria are as follows:

- Adults (≥18 years of age)
- For whom the physician in charge considers that NIV is indicated
- With a level of consciousness good enough to benefit from the musical intervention (Glasgow Coma Scale ≥12).

The non-inclusion criteria include:

- Patients with a classical contraindication to NIV
- Patients with severe hearing impairment
- Patients for whom a decision of withdrawal of life-sustaining therapies has been made, and with an estimated life expectancy of less than 48 hrs
- Patients included in another trial dealing with an ARF treatment strategy.

### Randomization

Eligible consecutive patients are randomly allocated to one of the three study arms. Randomization and concealment are achieved using a centralized, secure, computer-generated, interactive, web-response system accessible from each study center (CleanWEB, Telemedicine technologies S.A.S, Boulogne-Billancourt, France) [21]. The randomization is balanced by blocks of variable and undisclosed size and stratified on the center and the presence of a chronic respiratory insufficiency. Before randomization, the presence of the inclusion criteria and the absence of the non-inclusion criteria are verified and entered in the electronic case report form. In each ICU, patients are enrolled by the local physicians and a clinical research nurse and/or clinical research assistant. The randomization day is the study day 1.

### Blinding

For obvious reasons, this study cannot be blinded, either on the patient’s side (receiving or not receiving headphones, and listening or not listening to music during NIV sessions) or the caregiver’s side (helping the patient to choose the music and to adjust the headphones and the blinding mask). However, the evaluation of the primary endpoint is performed blindly from the treatment group according to the prospective randomized open blinded endpoint (PROBE) method [22]. Indeed, the primary endpoint is respiratory comfort after the 30 minutes of the first NIV session after randomization; this endpoint is assessed by an independent person who did not participate in the initiation of the NIV session and has no knowledge of the actual treatment received during the session. The organization of our ICUs allows requiring a blinded evaluator such as a nurse or a nursing assistant from another unit. The patient is told to not give any clue about the randomization arm during the evaluation. This same investigator will conduct assessments of the following NIV sessions; as far as possible, his/her independence with regard to the randomization group will be maintained in subsequent NIV sessions.

### Study interventions

**Fig. 1:**
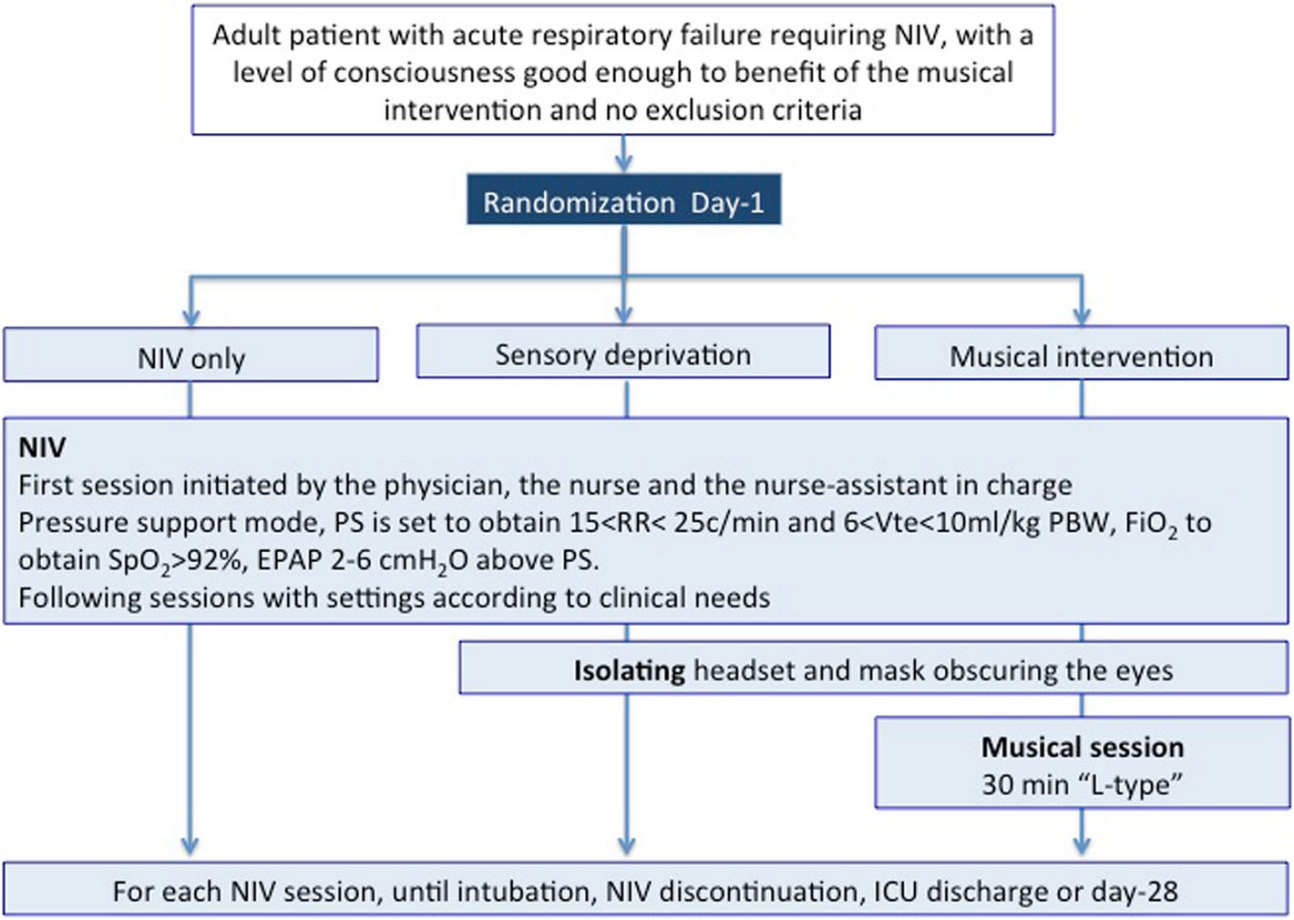
Study protocol and randomization arms. Pressure support is progressively increased in order to obtain a respiratory rate between 15 and 25 cycles per minute, an exhaled tidal volume of 6–10 ml/kg of predicted body weight, and the disappearance of signs of respiratory distress. Positive end expiratory pressure is set 2–6 cm H_2_O above pressure support and adjusted according to patient’s tolerance. FiO_2_ is set to obtain a minimal pulse oximetry of 92 %. *NIV* non-invasive ventilation, *PS* pressure support, *RR* respiratory rate, *Vte* exhaled tidal volume, *PBW* predicted body weight, *FiO*_*2*_ fraction of inspired oxygen, *SpO*_*2*_ peripheral capillary oxygen saturation, *EPAP* expiratory positive airway pressure, *ICU* intensive care unit

The study protocol and the three randomization arms are summarized on Fig. 1.

#### NIV conduction in all three groups

NIV is initiated according to standards of care and in line with each participating ICU’s practices. The detailed NIV protocol is given in Additional file 1. Briefly, masks are chosen according to the patient's morphology, and the physician in charge, along with the nurse and the nursing assistant, initiates the first session.

- Ventilator settings

Initial ventilator settings are chosen to maximize the patient's tolerance and minimize air leaks, in order to obtain a respiratory rate between 15 and 25 cycles per minute, an exhaled tidal volume of 6–10 ml/kg of predicted body weight, and the disappearance of signs of respiratory distress. FiO_2_ is set to obtain a minimal pulse oximetry of 92 %. The duration of NIV sessions is left to the discretion of the physician in charge, based on the patient’s needs.

- Subsequent NIV sessions

The subsequent NIV sessions are conducted by the nurse and nursing assistant according to a prespecified timeline: the physician in charge prescribes the length of NIV sessions and the number per day.

- Patient monitoring during NIV sessions

All patients undergo careful monitoring to detect and treat any complications related to ARF or NIV. Criteria for intubation are those in use in the participating ICUs [23].

#### Control group: “NIV only”

In this group, NIV is conducted as detailed above. Usually, the nurse and the nursing assistant provide psychological support and relaxation care to help the patient cope with NIV discomfort.

#### “Sensory deprivation” group

In this group, NIV is conducted similarly as for the former group but with the addition of sensory deprivation. Sensory deprivation consists of isolating participants from the noise and lights of the ICU:

- An insulating around-ear headphone (BOSE AE2®, the same as that used in the latter intervention arm) is placed on the patients’ ears by the unblinded caregivers, once the NIV mask has been fitted and the ventilator settings optimized.
- Applying a sleeping mask conceals the eyes. If the patient does not tolerate being blindfolded (e.g., due to claustrophobia), alternative solutions are suggested to the patient (such as closing his eyes or using a personal scarf) to make him feel at ease. The purpose of the visual deprivation is to avoid the patient being disrupted from the relaxation process because of visual distractions.

The headphones and mask will be left in place for 30 minutes. Data regarding tolerance and possible rejection of sensory deprivation will be collected.

After inclusion, all NIV sessions are conducted as detailed above until NIV discontinuation, day 28, or ICU discharge.

#### “Musical intervention" group

NIV is conducted as detailed above, including visual isolation. The musical intervention is initiated starting from the first NIV session that follows randomization.

**Fig. 2:**
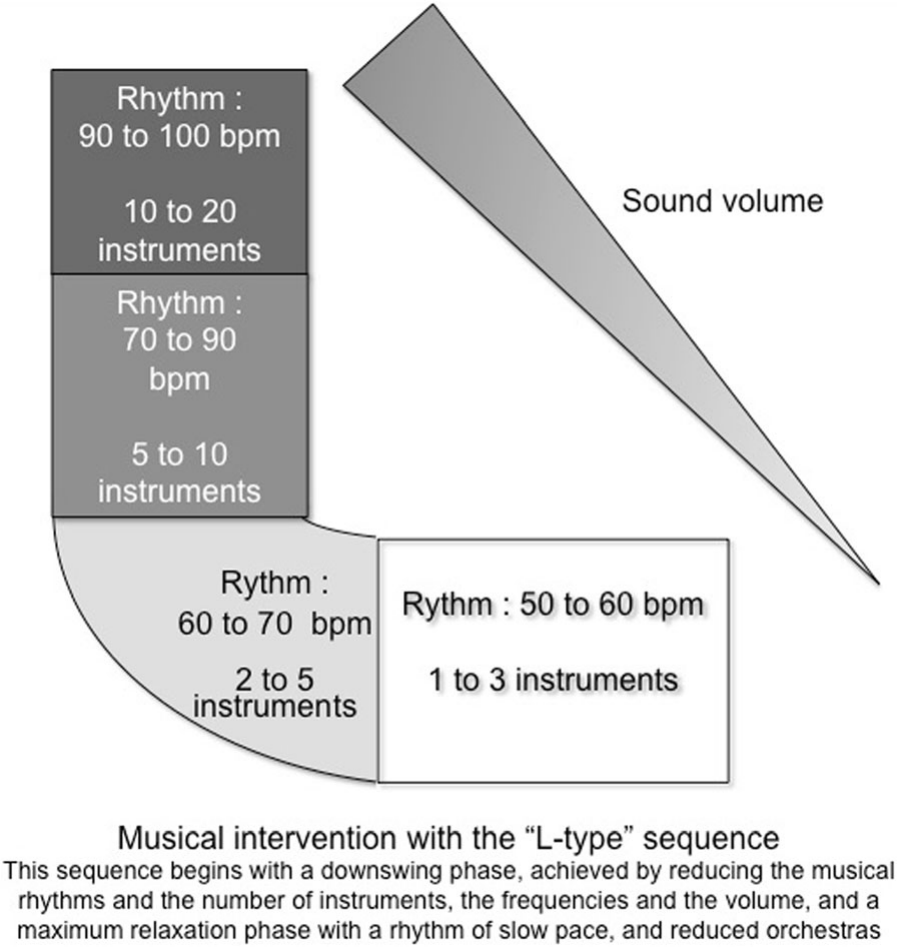
Musical intervention with the L-type sequence [24]. This sequence begins with a downswing phase, achieved by reducing the musical rhythms and the number of instruments, the frequencies, and the volume, and a maximum relaxation phase with a slow-paced rhythm and reduced orchestras (bottom of the L). *bpm* beats per minute

- Once the NIV mask has been fitted and the ventilator settings optimized, the trained nurse or nursing assistant sets up the headphones (BOSE AE2®), presents the tablet interface (Samsung Galaxy®), and shows the patient how to handle it.
- The patient’s musical tastes are determined by a caregiver-administered questionnaire, either to the patient or to her relatives if the patient is unable to express her musical choices (moderate impairment of consciousness). The patient therefore chooses the musical program, with MUSIC CARE^©^ software [19, 24, 25], according to her musical preferences, sets the volume level, and runs the musical session.
- The musical intervention session lasts 30 minutes and contains two phases, called an L-type sequence (Fig. 2) [24]; the downswing phase is achieved by reducing the musical rhythms starting at high tempos and a high number of instruments, gradually leading to slower tempos and reducing the number of instruments, the frequencies, and the volume. Then the patient is moved through a maximum relaxation phase with a slow-paced rhythm and reduced orchestras, resulting in maximum relaxation (bottom of the “L”).
- If the patient asks for more than 30 minutes of musical intervention, the session is extended towards a ”U-type” [25].

After inclusion until NIV discontinuation, day 28, or ICU discharge, all NIV sessions are performed according to this mode.

## Data collection and follow-up

Collected data at each time are summarized in the SPIRIT schedule of events shown in Table 1.

**Table 1:** Summary of collected data at each time point according to SPIRIT 2013 guidelines

			Study period							
	Enrollment	Allocation	Post-allocation — at each NIV session						NIV discontinuation, ICU discharge or day 28	Close-out Day 90
Time point			*t*_1_ beginning of NIV session	*t*_2_(*t*_1_ + 5 min)	*t*_3_(*t*_1_ + 30 min)	*t*_4_(*t*_1_ + 1 h)	*t*_*x*_^a^	End of each NIV session		
Enrollment:										
Eligibility screen	X									
Informed consent	X									
Allocation		X								
Interventions:										
Musical intervention										
Sensory deprivation										
Control group										
Assessments:										
*Baseline variables:* Demographic data and medical history, SAPS III, SOFA	X									
*Outcome variables:* Respiratory comfort, RASS			X	X	X	X	X	X		X
Respiratory and hemodynamic parameters		X	X	X	X	X	X	X		
*Ongoing therapies:* Catecholamine infusion, need for ETI, attempts to pull out NIV mask, headphones or sensory isolation, need for physical contention, sedative or anxiolytic treatments, NIV complication		X						X		
Health-related quality of life (HADS and SF-36 scores)		X								X
Length of ICU and hospital stay										X
Vital status, NIV satisfaction, comfort and associated trauma, IES-R for NIV									X	X
Decision to withhold life-sustaining therapies									X	

*NIV* non-invasive ventilation, *ETI* endotracheal intubation, *HADS* Hospital Anxiety and Depression Scale [28], *IES-R* Impact of Event Scale - Revised [31], *RASS* Richmond Agitation-Sedation Scale [30], *SAPS III* Simplified Acute Physiological Score III [26], *SF-36* Short Form-36 [29], *SOFA* Sequential Organ Failure Assessment [27]

^a^ *t*_*x*_ stands for *t*_1_ + 2, 3, 4, 6, 8, 12, 16, 20, 24 hours depending on the length of each NIV session

### At day 1 (baseline)

Demographic data and medical history, including the current clinical history with the reason for ICU admission, Simplified Acute Physiology Score III severity score [26], and Sequential Organ Failure Assessment score [27], are collected.

Treatments including NIV (and its settings), fluid therapy, catecholamine infusion, and endotracheal intubation are recorded. Laboratory tests include serum (and urine if diuresis is present) electrolyte levels determination, serum glucose level, urea and creatinine concentration, and arterial blood gas determination. Respiratory and hemodynamic parameters before the beginning of the NIV session (respiratory rate, exhaled tidal volume, PaO_2_/FiO_2_ ratio, heart rate, arterial pressure) are collected.

The research team administers to the patient, or to a proxy if the patient is not able to answer, the baseline Hospital Anxiety and Depression Scale (HADS) questionnaire [28] and Short Form-36 (SF-36) [29] in order to determine the baseline health-related quality of life.

### At each NIV session, until NIV discontinuation or day 28

The prescribed duration of the NIV session is recorded. Prior to each NIV session, immediately after NIV is correctly set (5 minutes), at 30 minutes, and at 1, 2, 3, 4, 6, 8, 12, 16, 20, 24 hours depending on the length of each NIV session, and at the end of the session, the following data are collected:

- Respiratory comfort assessed by a blinded evaluator (nurse from another unit) with the use of a numeric visual scale [14]
- The same respiratory and hemodynamic parameters as the initial ones
- Agitation assessed by Richmond Agitation-Sedation Scale [30].

For each NIV session, the need for physical contention or sedative or anxiolytic treatments is assessed and recorded. The tolerance of sensory isolation is recorded, as well as attempts to pull off the NIV mask or headphones. At the end of each session, data for its exact duration, the reason of its premature ending if appropriate, and the need for endotracheal intubation are collected. Furthermore, arterial blood gases are reported when these tests are performed as clinically indicated. The occurrence of nasal bridge ulcers or irritations is also recorded. For the “musical intervention” group, the type of music chosen and the feeling experienced during the session are recorded.

### At NIV discontinuation, ICU discharge, or day 28

Apart from endotracheal intubation, the need for tracheostomy during the ICU stay and vital status at ICU discharge are recorded. Patients are surveyed for numeric scaling of NIV satisfaction, NIV comfort, and NIV-associated trauma. The Impact of Event Scale - Revised (IES-R) [31] is applied to NIV.

### At day 90

Vital status is regularly updated for each patient until the end of the study. In order to determine the 3-months health-related quality of life, numeric scaling of NIV satisfaction, NIV comfort, and NIV-associated trauma, the IES-R applied to NIV, and the HADS questionnaire [28] and SF-36 [29] are determined and recorded by a blinded-to-randomization group research staff member through a phone survey of patients who are alive.

## Organization of the trial

### Funding/support

The Mus-IRA trial is sponsored by the Assistance Publique - Hôpitaux de Paris and supported by a grant of the French Ministry of Health dedicated to nursing care (Programme Hospitalier de Recherche Infirmière et Paramédicale 2013; PHRIP 13–453).

### Coordination and implementation of the trial

Each medical and paramedical team of the three participating ICUs was trained in the protocol and data collection using the electronic case report form (eCRF) during formal meetings prior to the start of screening and inclusion. The eCRF is developed with CleanWEB™, a centralized, secure, interactive, web-response system accessible from each study center, provided and managed by Telemedicine technologies.

Local physicians and clinical research assistants in each participating ICU are responsible for daily screening and inclusion of patients, compliance with protocol, availability of data requested for the trial, and completion of the eCRF. In accordance with French law, the eCRF and database were validated by appropriate committees (CCTIRS: Comité Consultatif sur le Traitement de l'Information en matière de Recherche dans le domaine de la Santé; CNIL: Commission Nationale de l'Informatique et des Libertés).

## Study endpoints

### Primary endpoint

The primary endpoint is the change in respiratory comfort before the initiation and after 30 minutes of the first NIV session after randomization. The respiratory comfort will be measured using a digital visual scale [14]. This scale is a 10-cm long ruler, shaped like an arrow. It is bounded on the left by “0: no respiratory discomfort” (the smaller base of the arrow) and on the right by “10: maximal respiratory discomfort” (the larger base of the arrow).

The participant marks the level of his/her perception of discomfort directly on the ruler.

### Secondary endpoints

Secondary endpoints are:

- The evolution of respiratory comfort during the first session of NIV evaluated before initiation, after 5 and 30 minutes, and at 1, 2, 3, 4, 6, 8, 12, 16, 20, 24 hours depending on the length of each NIV session, and at the end of the session
- The evolution of respiratory comfort evaluated at the same times in each subsequent session, measured using a digital visual scale [14]
- Changes in respiratory parameters during NIV sessions (respiratory rate, transcutaneous oxygen saturation, exhaled tidal volume) at 5 and 30 minutes and at 1, 2, 3, 4, 6, 8, 12, 16, 20, 24 hours depending on the length of each NIV session and at the end of the session
- Changes in cardiovascular parameters during NIV sessions (heart rate, arterial pressure) at 5 and 30 minutes and at 1, 2, 3, 4, 6, 8, 12, 16, 20, 24 hours depending on the length of each NIV session and at the end of the session
- The percentage of patients requiring endotracheal intubation (NIV failure) at the end of an NIV session
- The adequacy of the prescribed NIV session duration and its actual duration
- The number of sessions interrupted before the end of the prescribed time
- The percentage of patients requiring physical contention, sedative, or anxiolytic treatments during NIV sessions and ICU stay
- Anxiety/depression and health-related quality of life evaluated by HADS and SF-36 scores at baseline and after 3 months
- Post-trauma stress induced by NIV, measured with the IES-R scale immediately at discontinuation of NIV session and after 3 months of inclusion
- The overall assessment of NIV (in terms of discomfort, satisfaction, and trauma) at the discontinuation of NIV and at day 90.

## Statistical methods

### Sample size calculation

The assumptions used to calculate the number of participants for the study were determined from the article by Constantin et al*.* [14]. In that prospective, randomized, controlled trial assessing the "impact of sophrology on the tolerance of NIV sessions in patients with ARF," the difference in respiratory discomfort between patients treated with sophrology and the control group was 2.8, with a standard deviation of 2.5.

For our study, we considered the same standard deviation but a less optimistic difference for the comparison of the “sensory deprivation” group and the “musical intervention" group. Seventy-eight participants (26 participants per arm) should be included to obtain a power of 80 % to demonstrate a difference of 2 units of respiratory discomfort between the two groups, with an alpha risk of 5 % (bilateral formulation).

Three comparisons will be performed to assess the primary endpoint: a comparison between "musical intervention" and “sensory deprivation," a comparison between "musical intervention" and "NIV alone," and a comparison between "sensory deprivation" and "NIV only." If the three comparisons were each performed with a significance level of 5 %, the overall type I error rate would be far above the nominal level (14 %). To maintain an overall type I error rate of 5 % in a strong sense, we will apply a non-parametric Bonferroni-based chain procedure [32] for the analysis (as explained in the following section on statistical analysis), which implies reducing the significance level of two comparisons at 2.5 %. In order to maintain a power of 80 %, the number of planned participants was increased to 93 in total (31 per arm).

### Total planned sample size

To take into account a potential loss to follow-up of about a 5 % rate for the primary endpoint, we expect to randomize a total of 99 participants (33 per arm).

### Statistical analysis

A flow chart will describe the number of eligible patients and the number of patients actually included (in total and per arm). For each group and at each assessment date, qualitative variables will be described in terms of number and percentage, and quantitative variables in terms of number, mean, and standard deviation. Quantitative variables with skewed distribution will be presented in terms of median and interquartile range (25th–75th percentile).

#### Primary endpoint

The change in respiratory comfort at initiation and after 30 minutes of the first NIV session will be analyzed in the intention-to-treat population. For each patient, the variation in respiratory comfort will be calculated by the difference between the measurement at “30 minutes” and "before." This change will then be compared between the treatment groups, based on a Student’s *t* test. If the test application conditions are not met, a Wilcoxon test will be applied.

**Fig. 3:**
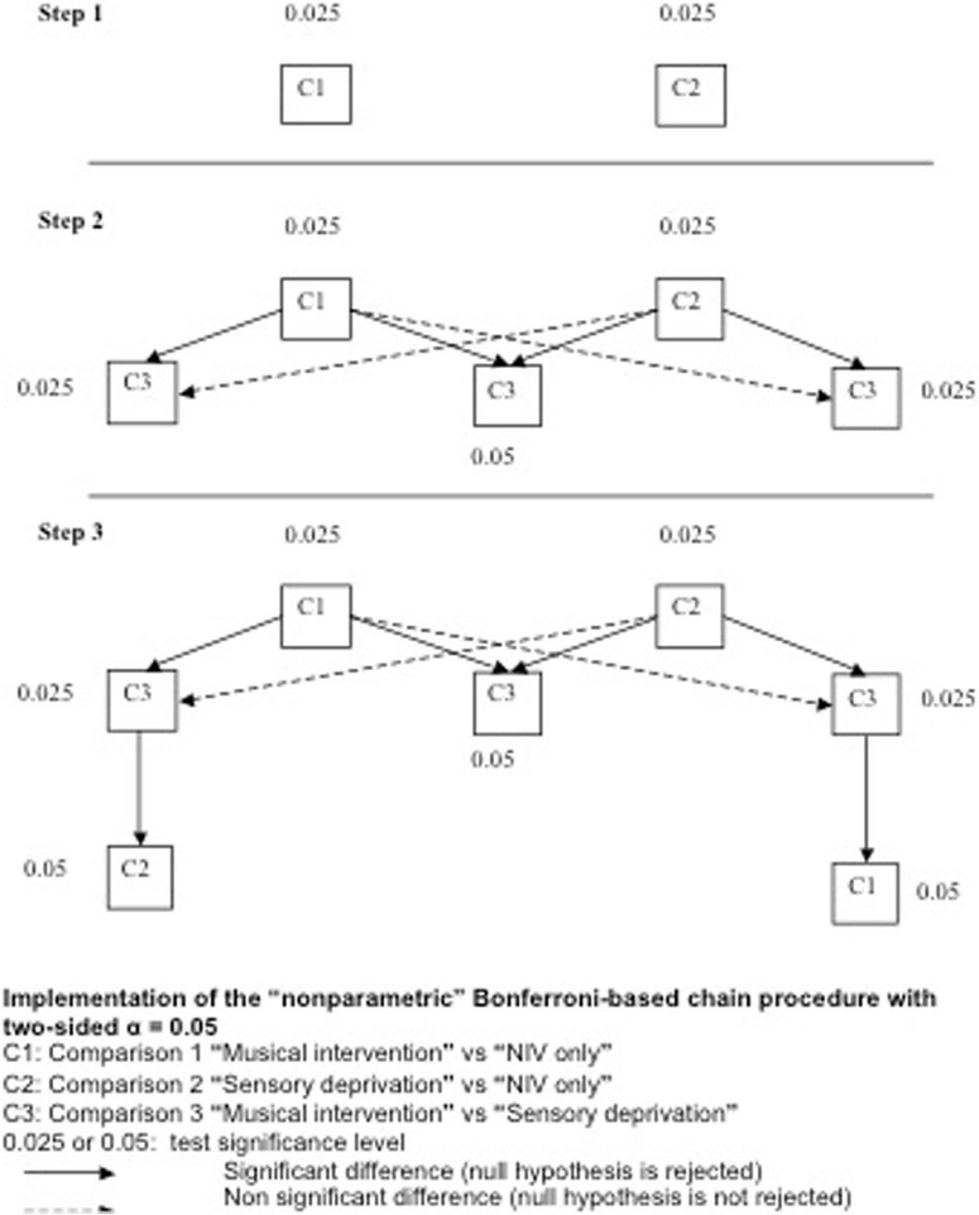
Hierarchical test procedure used for the primary endpoint analysis

Because three parallel groups are included in the study, three comparisons are possible: "musical intervention" and "NIV only" (comparison 1), "sensory deprivation" and "NIV only" (comparison 2), and "musical intervention" and "sensory deprivation" (comparison 3). These comparisons will be performed in a pre-established hierarchical test procedure (see Fig. 3), in order to maintain the alpha risk of 5 % in a strong sense:

- Step 1: We will perform comparisons 1 and 2, both at 2.5 % (bilateral) alpha risk.
- Step 2: In the event of non-significance of the two previous tests, we will stop the test procedure and conclude the negativity of the trial; in the case of statistical significance of both previous tests, we will perform comparison 3 at 5 % (bilateral) alpha risk; in the case of statistical significance of only one of the two previous tests, we will perform comparison 3 at 2.5 % (bilateral) alpha risk.
- Step 3: If comparison 3 was performed at 2.5 % alpha risk and is significant, the non-significant comparison performed in step 1 may be performed again at an alpha risk of 5 % (Fig. 3).

#### Other analyses

All secondary analyses will be performed in the intention-to-treat population (all randomized patients) and also in the per protocol population (patients with no major deviation of the protocol) at a bilateral 5 % alpha risk.

Unless otherwise specified, categorical variables will be compared by a chi-squared test or Fisher test as appropriate. Continuous variables will be compared by a Student's *t* test or Wilcoxon test as appropriate (when two groups are compared) and by an analysis of variance (ANOVA) or Kruskal-Wallis test as appropriate (when more than two groups are compared).

##### Adjusting the analysis of the primary endpoint for potential confounders

The primary endpoint analysis will be adjusted on the stratification factors (centers and underlying chronic respiratory disease) and on potential confounders using a multivariate analysis (linear regression model). Potential confounders will be: a prior chronic respiratory disease; a PaO_2_/FiO_2_ < 200 mmHg; a prior psychiatric disease.

##### Primary endpoint analysis in the per protocol population

The change in respiratory comfort at initiation and after 30 minutes of the first NIV session between the groups will also be compared in the per protocol population defined previously (this comparison does not constitute the primary analysis of the study).

##### Evolution of respiratory comfort during NIV sessions

The number of measures of respiratory comfort for each patient will be variable, since the duration and number of NIV sessions is at the discretion of the physician. The data structure is complex and has three levels:

- Level 1: respiratory discomfort measures
- Level 2: patients
- Level 3: centers.

For this type of (repeated) data, it is essential to take into account correlations between measurements from the same patient. A linear mixed effects model (including fixed and random effects) will be used to model the effect of the three interventions on the evolution of respiratory comfort during NIV sessions. This model will include:

- Fixed effects
  - ○ The variable "time"
  - ○ The randomization group (categorical variable with three different levels) and its interaction with time
  - ○ Stable characteristics over time (see potential confounders described above)
  - ○ Characteristics which vary over time: the number of NIV sessions
- Random effects ("subject effect").

The need for a model with random intercept and slope (versus random intercept only) will be assessed at the time of the analysis with a likelihood ratio test. An appropriate modeling of time will be performed if its effect is not linear.

Correlations between patients from the same center could also be taken into account by the inclusion of a "random center effect" (level 3). However, such a mixed model may be unsuitable in practice because of the limited number of clusters (only three participating centers). In this setting, it is preferable to include a fixed center effect [33], and this is the solution that will be implemented in this analysis.

##### Evolution of respiratory and hemodynamic parameters during NIV sessions

This analysis will be conducted according to the same principles as for the analysis of the evolution of respiratory comfort.

##### Adequacy of prescribed and observed NIV sessions duration

For this analysis, we will first calculate for each patient the ratio of the sum of the observed durations of NIV sessions and the sum of the prescribed durations. This ratio will reflect the compliance of each patient with the prescription. Compliance will be compared between groups by the usual tests for comparing a continuous outcome.

##### Number of interrupted sessions before the end of the prescribed duration

The number of interrupted sessions is a count outcome variable. The comparison groups will therefore be based on a Poisson regression model, introducing the randomization group as an explanatory variable and an "offset" variable (whose coefficient is not estimated and is fixed to 1) representing the number of prescribed sessions for each patient.

##### Percentage of patients requiring physical restraints or administration of anxiolytics or sedatives during NIV sessions, percentage of intubated patients

These percentages will be compared between groups by the usual tests for comparing proportions.

##### SF-36, HADS scores, IES-R scores, and overall assessment of NIV between inclusion and day 90

The evolution of these scores between baseline and day 90 (difference between the measurement at 3 months and inclusion) will be compared between groups by the usual tests comparing continuous variables.

#### Missing data

First, all analyses will be performed without taking into account missing values. Second, a sensitivity analysis will be performed using multiple imputations to replace missing values where appropriate for primary and all secondary outcomes [34]. We will create 10 copies of the dataset, with the missing values replaced by imputed values, based on observed data including outcomes and baseline characteristics of participants. Each dataset will be analyzed using standard statistical methods, and the results from each dataset will be pooled into a final result using Rubin’s rule.

#### Software

The analyses will be performed using R software (R Foundation for Statistical Computing, Vienna, Austria, http://www.R-project.org/) 3.0 or later or SAS version 9.2 or later.

## Safety considerations

In the current study there are no anticipated risks or inconveniences. For this reason, neither safety interim analysis nor stopping rules are planned. Any unexpected medical event after inclusion (adverse events, AEs) will be recorded on the individual CRFs. Unexpected serious adverse events (SAEs) will be reported to the sponsor immediately, and all participating centers will be informed by the sponsor (Department de la Recherche Clinique - Assistance Publique - Hôpitaux de Paris).

## Discussion

In this randomized controlled trial, we expect an improvement of NIV tolerance and efficacy thanks to a simple, easily implemented, non-pharmacological intervention.

In everyday life, medical teams strive to improve patients’ care, and patient centeredness is crucial to quality of care [35]. Intolerance is one of NIV’s major drawbacks and has been associated with a higher risk of intubation [7–9]. In the study of Delclaux et al. of NIV patients with hypoxemic ARF [8], intolerance was responsible for 14 % of NIV failures, leading to intubation in a third of them, a finding shared by others [9]. In order to improve NIV tolerance, ventilators have been bench-marked, and their performances rated [36–38]. Likewise, physicians have focused their efforts on assessing appropriate humidification devices [39] and ventilator-patient interface types [40], and on reducing associated leaks [41]. Beyond these technical aspects, however, patient-centered outcomes may have been overlooked and must be taken into account.

This trial involves patient-centered care at two different levels. First, the patient is active in choosing the desired music type, which may maximize his/her adherence to the music intervention, as suggested by Chlan et al. with the use of a music preference assessment questionnaire [17]. Second, to evaluate music intervention efficacy, we defined a patient-reported measure as the primary outcome (variation of dyspnea discomfort) and as secondary outcomes (anxiety/depression and health-related quality of life at baseline and at day 90; post-trauma stress induced by NIV, at discontinuation of NIV session, and at day 90; the assessment of NIV discomfort, satisfaction, and trauma).

We have chosen to run this trial in three units that already provided patient-centered care, in particular regarding relatives’ visitation hours, patients’ comfort needs, and involvement of patients and their relatives in the decision-making process.

Music therapy has shown its beneficial effects on patient anxiety, pain [24], and physiological events (heart rate, blood pressure) either outside [15, 42–44] or inside the ICU [16–20]. These studies showed that music therapy is feasible in the ICU, although only a small proportion of invasively ventilated patients (5.3 %) might benefit from such an intervention [17]. As such, music effects have never been assessed during NIV, a ventilator mode increasingly used in the ICU [45]. Therefore, one may surmise that many more patients will be affected by this intervention. Given that anxiety is one of NIV’s major drawbacks, our aim is to decrease potential stressful feelings that might be induced by NIV with the music therapy. This has been shown in the ICU: Han et al. [18] showed that a single music session significantly decreased anxiety (measured by the Spielberger State/Trait Anxiety Inventory) in patients invasively ventilated, compared to those who did not receive music. Likewise, Chlan et al. showed a significant effect of patient-directed music therapy on reducing anxiety (measured by a visual analog scale) compared with usual care during ICU stays in patients being ventilated [17].

It has been shown that post-ICU symptoms of anxiety, depression, or post-traumatic stress disorder (PTSD), especially in NIV patients [46], might occur up to 90 days after an ICU stay. Unfortunately, these symptoms were not assessed after this time in previous studies of music intervention, and never in NIV patients. In our trial, we plan to evaluate these symptoms and to find if a punctual intervention (i.e., 30 minutes of music intervention during NIV sessions) can diminish such post-ICU symptoms. Short- and long-term assessment of NIV-related PTSD seems necessary to determine the length of action of the interventions being tested.

Chlan et al. evidenced that music administration led to significantly less anxiety and sedation intensity compared to usual care in ventilated patients. But this was not evidenced when comparing usual care to noise-canceling headphones. We therefore decided to build a three-arm trial in order to distinguish the effects of the music and of the isolation of the noise and the light of the ICU. As ICU patients report noise as the main source of discomfort [47], we naturally question in our trial the role of noise isolation and of music in NIV (and ICU) tolerance, and therefore tested the “sensory deprivation” strategy.

To conclude, our trial tests an original non-pharmacologic, simple, and toxin-free therapy to improve one of the most stressful situations in the ICU. We deliberately choose patient-centered outcomes to assess this patient-centered intervention.

## Trial status

Enrollment is ongoing. Inclusions started on May 2015 and are expected to be completed in May 2016.

## Additional files

Additional file 1: NIV protocol. (DOCX 103 kb) Additional file 2: SPIRIT guidelines checklist. (DOC 121 kb)

## Abbreviations

ARF: Acute respiratory failure
CCTIRS: Comité Consultatif sur le Traitement de l'Information en matière de Recherche dans le domaine de la Santé
CE SRLF: Commission d’Ethique de la Société de Reanimation de Langue Française
CNIL: Commission Nationale de l'Informatique et des Libertés
eCRF: Electronic case report form
FiO_2_: Fraction of inspired oxygen
HADS: Hospital Anxiety and Depression Scale
IES-R: Impact of Event Scale - Revised
ICU: Intensive care unit
NIV: Non-invasive ventilation
PaO_2_: Arterial oxygen partial pressure
PHRIP: Programme Hospitalier de Recherche Infirmière et Paramédicale
SAPS: Simplified Acute Physiology Score
SF-36: Short Form-36
SOFA: Sequential Organ Failure Assessment
