# Supplementary material for: Effect of a musical intervention on tolerance and efficacy of non-invasive ventilation in the ICU: study protocol for a randomized controlled trial (MUSique pour l’Insuffisance Respiratoire Aigue - Mus-IRA)
Source: Trials. 2016 Sep 13;17(1):450. doi: 10.1186/s13063-016-1574-z (PMC5020479; doi:10.1186/s13063-016-1574-z)
Supplement: Additional file 1: — NIV protocol. (DOCX 103 kb) [file 13063_2016_1574_MOESM1_ESM.docx]

Effect of a musical intervention on tolerance and efficacy of non-invasive ventilation in the ICU: study protocol for a randomized controlled trial (MUSique pour l’Insuffisance Respiratoire Aigue - Mus-IRA) – Supplementary material

Jonathan Messika MD^1,2,3^, David Hajage MD^4,5,6^, Nataly Panneckoucke RNA^1^, Serge Villard RN^1^, Yolaine Martin RN^1^, Emilie Renard RN^1^, Annie Blivet RN^1^, Jean Reignier MD, PhD^7^, Natacha Maquigneau RN^7^, Annabelle Stoclin MD^8^, Christelle Puechberty RN^8^, Stéphane Guétin PhD ^9^, Aline Dechanet CRA^6^, Amandine Fauquembergue CRA^6^, Stéphane Gaudry MD^1,4,5^, Didier Dreyfuss MD^1,2,3^, and Jean-Damien Ricard MD, PhD^1,2,3^.

^1^ AP-HP, Hôpital Louis Mourier, Medical-Surgical Intensive Care Unit, 178 rue des

Renouillers, F-92700, Colombes, France

^2^ Univ Paris Diderot, Sorbonne Paris Cité, IAME, UMRS 1137, F-75018 Paris, France

^3^ INSERM, IAME, U1137, F-75018 Paris, France

^4^ Univ Paris Diderot, Sorbonne Paris Cité, ECEVE, UMRS 1123, F-75010 Paris, France

^5^ INSERM, ECEVE, U1123, F-75010 Paris, France

^6^ INSERM, CIC-EC 1425, UMR 1123, Paris, France; Assistance Publique–Hôpitaux de

Paris, Hôpital Louis Mourier, Département d’Epidémiologie et Recherche Clinique, Paris,

France, Univ. Paris Diderot, UMR 1123, Sorbonne Paris Cité, Paris, France;

^7^ Medical-Surgical Intensive Care Unit, District Hospital Center, La Roche-sur-Yon, France

^8^ Medical-Surgical Intensive Care Unit, Institut Gustave Roussy, Villejuif, France

^9^ Department of Neurology, Inserm U1061, Gui de Chauliac University Hospital and Montpellier School of Medicine, Montpellier, France.

**NIV protocol**

In all three groups, NIV is initiated as follows, according to standards of care, and in line with each participating ICU’s practices. Patients have continuous electrocardiographic and percutaneous oxygen saturation monitoring. NIV is performed using the NIV function of ICU ventilators. Size and type of masks (nasal, oral, or full-face) are chosen according to the patient's morphology, thus optimizing patient-interface and promoting patient comfort. The physician in charge, along with the nurse and the nurse-assistant initiate the first session. The mask is correctly and carefully adjusted, with the help of head straps, and an hydrocolloid sheet is applied over the nasal bridge to limit pressure ulcers. Initial ventilator settings are chosen to maximize patient's tolerance and minimize air-leaks. Pressure support is progressively increased in order to obtain a respiratory rate between 15 and 25 cycles per minute, an exhaled tidal volume between 6-10 ml/kg of predicted body weight, and the disappearance of signs of respiratory distress. Positive end expiratory pressure is set 2-6 cmH_2_O above pressure support, and adjusted according to patient’s tolerance. FiO_2_ is set to obtain a minimal pulse oximetry of 92%. Duration of NIV sessions is left at the physician in charge discretion, based on patients’ needs. Between NIV sessions, low or high flow oxygen therapy is administered according to patients’ needs to obtain a minimal pulse oximetry of 92%.

The nurse and nurse assistant can conduct the ensuing NIV session, according to the medical prescription.

All patients undergo careful monitoring to detect and treat any complications related to ARF or NIV. Criteria for intubation are those in use in the participating ICUs such as inability to correct dyspnea, to tolerate the NIV or to maintain a PaO_2_ above 65mmHg with a FiO_2_ of 0.6 or greater; inability to manage tracheal secretions; neurological failure or seizures; hemodynamic instability [1]

Reference

1. Antonelli M, Conti G, Rocco M, Bufi M, De Blasi RA, Vivino G, et al. A comparison of noninvasive positive-pressure ventilation and conventional mechanical ventilation in patients with acute respiratory failure. N. Engl. J. Med. 1998;339:429–35.

.
